# Leptospirosis—Improving Healthcare Outcomes for a Neglected Tropical Disease

**DOI:** 10.1093/ofid/ofaf035

**Published:** 2025-02-10

**Authors:** Claudia Muñoz-Zanzi, Anou Dreyfus, Umaporn Limothai, Walker Foley, Nattachai Srisawat, Mathieu Picardeau, David A Haake

**Affiliations:** School of Public Health, University of Minnesota, Minneapolis, Minnesota, USA; Section of Epidemiology, Vetsuisse Faculty, University of Zurich, Zurich, Switzerland; Center of Excellence in Critical Care Nephrology and Division of Nephrology, Department of Medicine, Faculty of Medicine, Chulalongkorn University, Bangkok, Thailand; Excellence Center for Critical Care Nephrology, King Chulalongkorn Memorial Hospital, Bangkok, Thailand; Ohio State College of Medicine, Ohio State University, Columbus, Ohio, USA; Center of Excellence in Critical Care Nephrology and Division of Nephrology, Department of Medicine, Faculty of Medicine, Chulalongkorn University, Bangkok, Thailand; Excellence Center for Critical Care Nephrology, King Chulalongkorn Memorial Hospital, Bangkok, Thailand; French National Reference Center for Leptospirosis, WHO Collaborating Center for Reference and Research on Leptospirosis, Institut Pasteur, Université Paris Cité, Biology of Spirochetes Unit, Paris, France; Veterans Affairs Greater Los Angeles Healthcare System, Los Angeles, California, USA; Department of Internal Medicine, David Geffen School of Medicine, University of California, Los Angeles, California, USA

**Keywords:** leptospirosis, neglected diseases, bacterial zoonoses, one health, global health

## Abstract

Leptospirosis is a globally distributed zoonotic disease transmitted from animal reservoirs to humans. It is particularly common in tropical regions of Africa, Asia, and Central and South America during heavy rainfall when bacterial spirochetes are released from soil into areas of flooding. Despite causing >1 million severe cases, 58 900 deaths, and 2.9 million disability-adjusted life-years annually—exceeding established neglected tropical diseases—leptospirosis remains underrecognized as a neglected tropical disease. It affects occupational groups like farmers due to high prevalence in livestock and is spread by rodents in urban settings that have poor sanitation and infrastructure. Although effectively treated with inexpensive antibiotics, neglect of leptospirosis research and development has led to a lack of awareness and unavailability of preventive and diagnostic approaches. This review covers the geographic prevalence, disproportionate impacts on marginalized communities, and opportunities for improving social, economic, and healthcare burdens for patients with leptospirosis.

## Teaser

Leptospirosis is a neglected tropical disease with important global health economic impacts that would respond well to investments in awareness, surveillance, reporting, and rapid diagnostics to facilitate early treatment with simple and effective antimicrobial therapy.

Leptospirosis is a widespread yet neglected tropical disease predominantly affecting resource-limited settings. Caused by bacterial spirochetes belonging to the genus *Leptospira*, it is transmitted to humans through contact with soil or water contaminated by the urine of infected animals, typically rats, and other synanthropic rodents, livestock, and many other mammals. Once released into the environment, most pathogenic leptospires can survive for extended periods and may multiply under suitable conditions [1]. Warm, humid climates with heavy rainfall and inadequate housing infrastructure heighten the risk, particularly where flooding occurs. The early symptoms of leptospirosis—fever, muscle pain, and headache—mimic other febrile illnesses like dengue and malaria, complicating diagnosis. The disease can also present atypically as aseptic meningitis, myocarditis, or pulmonary hemorrhage. Without timely treatment, a significant number of patients require intensive care because of multiorgan dysfunction involving the liver, kidney, lungs, and/or central nervous system [2]. Severe cases have a fatality rate of 5%–15% [3], which is even higher among patients with altered mental status [4] or pulmonary hemorrhage [5]. There is a growing recognition of the long-term health issues experienced by survivors [6, 7]. Surviving patients remain at risk of chronic postleptospirosis issues, including fatigue, myalgia, malaise, persistent headaches, and visual disturbances [8]. Several studies suggest a potential link between leptospirosis and chronic renal and neurological sequelae [7–13]. Marginalized communities, with limited healthcare access, bear the greatest burden, leading to delayed care and increased mortality risk. Those affected may suffer a loss of income because of illness and/or long-term disability, whereas communities face reduced productivity and increased healthcare costs [14, 15]. Leptospirosis also affects domestic animals, incurring significant economic losses in livestock from decreased productivity, reproductive losses (eg, abortions in cattle and pigs), and increased veterinary costs. Early diagnosis is vital for effective treatment and preventing severe complications. However, limited access to diagnostic tests and reliance on clinical suspicion hinder timely intervention. Educating healthcare professionals about the local disease epidemiology and recognizing early signs of leptospirosis can also improve early diagnosis and reduce disease severity [16, 17].

Neglected tropical diseases (NTDs) are conditions that impose severe health, social, and economic burdens, particularly in disadvantaged rural and urban areas of tropical regions [18]. The World Health Organization (WHO) defines NTDs as “ancient diseases of poverty that impose a devasting human, social, and economic burden on more than 1 billion people worldwide, predominantly in tropical and subtropical areas among the most vulnerable, marginalized populations” [19]. NTDs are often neglected in terms of research, funding, and public health interventions, but the premise is that increased awareness and investment can significantly reduce their impact and improve healthcare outcomes [20]. Leptospirosis has been widely recognized as meeting the criteria for an NTD, with multiple journal publications providing evidence of its status as such [18, 21–28]. Since the launch of *PLoS Neglected Tropical Diseases* in 2007, leptospirosis has been included as a major NTD within the journal's scope. However, despite this recognition and substantial evidence of its impact, leading public health organizations—including the WHO, the Pan American Health Organization, and US Federal Drug Administration—have yet to prioritize leptospirosis by officially adding it to their NTD lists. This oversight is particularly striking given that leptospirosis contributes to a greater healthcare burden in tropical regions than some diseases already classified as NTDs. For instance, leptospirosis is estimated to be responsible for 1.03 million severe cases and approximately 58 900 deaths and 2.9 million disability-adjusted life-years (DALYs) lost annually—figures that exceed those associated with other recognized NTDs such as lymphatic filariasis, rabies, and dengue [9, 23, 28, 29]. A recent study calculated the productivity cost of DALYs lost because of leptospirosis estimating the global annual productivity loss at $29.3 billion, which could be as high as $52.3 billion, with the highest economic burden affecting the Asia-Pacific region [30].

This review summarizes the evidence that leptospirosis is an NTD, including amenability to interventions likely to improve healthcare outcomes for patients. Effective prevention and control rely on a comprehensive One Health approach that addresses the socioeconomic and environmental factors such as improved sanitation to reduce rodent infestations, improved infrastructure to reduce flooding, and public education about exposure risks, while improving access to healthcare, diagnosis, treatment, and prevention in high-risk communities. Investment in basic healthcare services, strengthening surveillance systems, and promoting research and commercial development for better diagnostic tools and vaccines are crucial steps in mitigating the disease's impact on vulnerable populations. Addressing the problem of leptospirosis is a matter of social justice and health equity because it disproportionately affects those living in poverty.

## GEOGRAPHIC PREVALENCE

Tropical and subtropical regions—such as South and Southeast Asia, the Caribbean, parts of Africa, Latin America, and Oceania—experience significantly higher incidence and mortality rates compared to drier, colder areas [9, 29]. This elevated rate is due to a mix of environmental, socioeconomic, and infrastructural factors, alongside the disease's complex dynamics in these regions. The warm, humid climate of the tropics, combined with heavy precipitation, creates ideal conditions for the survival and transmission of *Leptospira* spp. The rainy season, particularly during monsoons, hurricanes and other major weather events, often coincides with increased cases and outbreaks [31–36], as heavy rains facilitate the dispersal of leptospires from soil into surface waters [3, 4, 31, 37–40]. Flooding amplifies the risk by exposing individuals to contaminated water, leading to large-scale outbreaks across high- and low-resource areas in rural and urban settings. Analysis of long-term leptospirosis case data in New Caledonia showed that climatic factors including El Niño Southern Oscillation and meteorological conditions were predictive of leptospirosis outbreaks [41].

Climate change and resulting increase in episodes of intense rainfall and flooding are expected to significantly impact the incidence and geographic distribution of leptospirosis. The Intergovernmental Panel on Climate Change reported an increase in the global mean surface temperature of approximately 1.09 °C between 2011 and 2020, with projected increases of 0.2 °C per decade, potentially reaching 1.4–4.4 °C by 2100 [42]. The Intergovernmental Panel on Climate Change predicts that rising global temperatures and more frequent extreme precipitation events will increase the burden of waterborne zoonotic diseases such as leptospirosis in many regions and could potentially expand the areas where infections are prevalent [43]. Climate-driven models demonstrate that both temperature and precipitation are highly predictive of the seasonal dynamics of leptospirosis in tropical regions, with some areas potentially experiencing increased incidence because of climate change [35, 44, 45]. Modeling also suggests that the burden of leptospirosis is significantly underappreciated in some areas [45]. The intersection of climate change effects (temperature rise, flooding and natural disasters, mass migration and poverty, and public health strain) with inadequate health and sanitation infrastructure combine to increase risk for leptospirosis. Although some countries are shifting toward climate adaptation financing, less than 0.5% of total climate finance is allocated for strengthening health systems, and even less is devoted to research and disease control in regions where climate change is expected to most significantly impact human health [46].

Agricultural activities and contact with livestock and rodents are strongly associated with an increased risk of leptospirosis in tropical regions. Occupations such as fish farming, rice cultivation, and work on sugar cane or banana plantations involve frequent contact with potentially contaminated soil and water ​ [47]. These areas often serve as feeding grounds for rodents that shed leptospires, creating a high-risk environment. The activities often occur in wet, muddy conditions that are conducive to bacterial survival and transmission. In addition to occupational risks, recreational activities like swimming in natural bodies of water also pose a threat [48].

As shown in Table 1, Oceania and South and Southeast Asia are considered to have the highest global incidence of leptospirosis. Incidence is particularly well documented in Sri Lanka, where hospital admissions for leptospirosis are estimated at 52.1 patients per 100 000 population, and the country reports >700 deaths annually—more than double the fatalities attributed to dengue fever [49]. A recent study found that 10% of sepsis in South India is due to leptospirosis [50]. Other high-burden countries include Thailand, Indonesia, and the Philippines, where agricultural activities, such as rice farming, combined with intense rainy seasons, facilitate the transmission of *Leptospira* from animal reservoirs to humans through contaminated floodwaters [9]. In the Federated States of Micronesia, there was a 20.4% incidence of acute leptospirosis among patients presenting with febrile illness [51]. In Malaysia, studies have shown a 72.5% seroprevalence of leptospirosis antibodies among cattle farmers in the northeastern region, with a 12.1% prevalence of pathogenic *Leptospira* detected in the environment on cattle farms [52].

**Table 1.** Estimated Annual Incidence and Disease Burden of Leptospirosis by Region

Regions	Incidence/100K^a^	Mortality/100K^a^	DALYs/100K^b^
Oceania	150.7	9.6	515
Southeast Asia	55.5	3.0	137
Caribbean	50.7	2.9	127
East sub-Saharan Africa	25.7	1.9	106
Central America	15.8	0.7	33
Tropical Latin America	13.5	0.7	31
Southern Latin America	3.9	0.2	8.0
Western Europe	3.9	0.2	7.1
North America	3.6	0.2	7.3
Southern Africa	3.4	0.3	18

Abbreviation: DALY, disability-adjusted life-year.

^a^Costa et al [9].

^b^Torgerson et al [29].

Leptospirosis is endemic throughout Latin America and the Caribbean, with greater incidences in the tropical and subtropical countries. Investigations of the role of leptospirosis in the local etiology of acute undifferentiated febrile illness showed variability by location. The percent of patients with acute undifferentiated febrile illness and leptospirosis has been reported to be 4% in Puerto Rico [53], 6% in Nicaragua [54], 5% and 9% in Peru [55, 56], 13% in Ecuador [57], and 14% in Colombia [58], which highlights the likely underestimation of reported data and the importance of considering leptospirosis among routine clinical management of fever patients [59]. Latin America and the Caribbean is the world's second most disaster-affected region after Asia and the Pacific and floods are the most common disaster. Frequent leptospirosis outbreaks of varying magnitude are reported following heavy rains, hurricanes, and natural disasters in tropical and subtropical countries [60, 61].

Leptospirosis is widespread in Africa with several studies documenting infection among various reservoir hosts, including rodents, small ruminants, pigs, and cattle ​ [62–65]. The incidence of leptospirosis in Tanzania was estimated to range from 75 to 102 cases per 100 000 people annually [66] and the prevalence of acute human leptospirosis ranged from 2.3% to 19.8% in hospital patients with febrile illness in 11 studies in various African countries [66]. Occupational risk factors have been identified among rural, pastoralist communities with close contact with livestock and rice farming in Tanzania [63, 67, 68] and abattoir workers in South Sudan showed serologic evidence of exposure [65]. A recent outbreak in Tanzania in 2022, involving 20 confirmed cases and 3 deaths [69], was associated with agricultural practices, urbanization, wildlife encroachment, and poor sanitation. During this outbreak, leptospirosis had only been diagnosed after exclusion of other diseases causing hemorrhagic fevers, indicating the low awareness and diagnostic challenges for leptospirosis. In the Democratic Republic of Congo, leptospirosis has been recognized as a neglected cause of fever and jaundice, particularly affecting young men in the slums of Kinshasa during the rainy season [70]. Despite its significance, leptospirosis in Africa remains underresearched and underreported.

## DISPROPORTIONATE IMPACT ON MARGINALIZED COMMUNITIES

Leptospirosis disproportionately affects marginalized communities [71–74] because of interconnected factors that lead to increased exposure to a contaminated environment. Rapid and uncontrolled urbanization in areas with deficiencies in sanitation and housing structural integrity create environments conducive to dense rodent populations [73] resulting in high levels of exposure to pathogenic leptospires. Globally, 1 billion people live in urban slums (projected to double by 2050), which present particular risks when inadequate drainage and sewer systems lead to widespread contamination during heavy rains or flooding [73, 75]. Prospective studies conducted in Salvador's slum communities in Brazil, revealed how inadequate infrastructure and extreme rainfall events combine to create heightened risks of peridomestic environmental contamination with pathogenic leptospires [76].

In the most impacted communities, limited healthcare resources and access to diagnostics is a major contributor to the underrecognition, underdiagnosis, and underreporting of leptospirosis [75]. Although significant advancements have been made in leptospirosis diagnostic research and development, most diagnostic testing demand comes from marginalized communities, which often cannot afford expensive diagnostic products. Consequently, pharmaceutical and medical device companies have little financial motivation to commercialize leptospirosis diagnostics. Compounding this problem, many tropical countries do not include leptospirosis in their reportable disease surveillance systems, making global estimates for morbidity and mortality inaccurate and biased [9]. Lack of awareness hampers efforts to secure funding and policy support for research, surveillance, control, and prevention initiatives [28].

**Figure 1.**
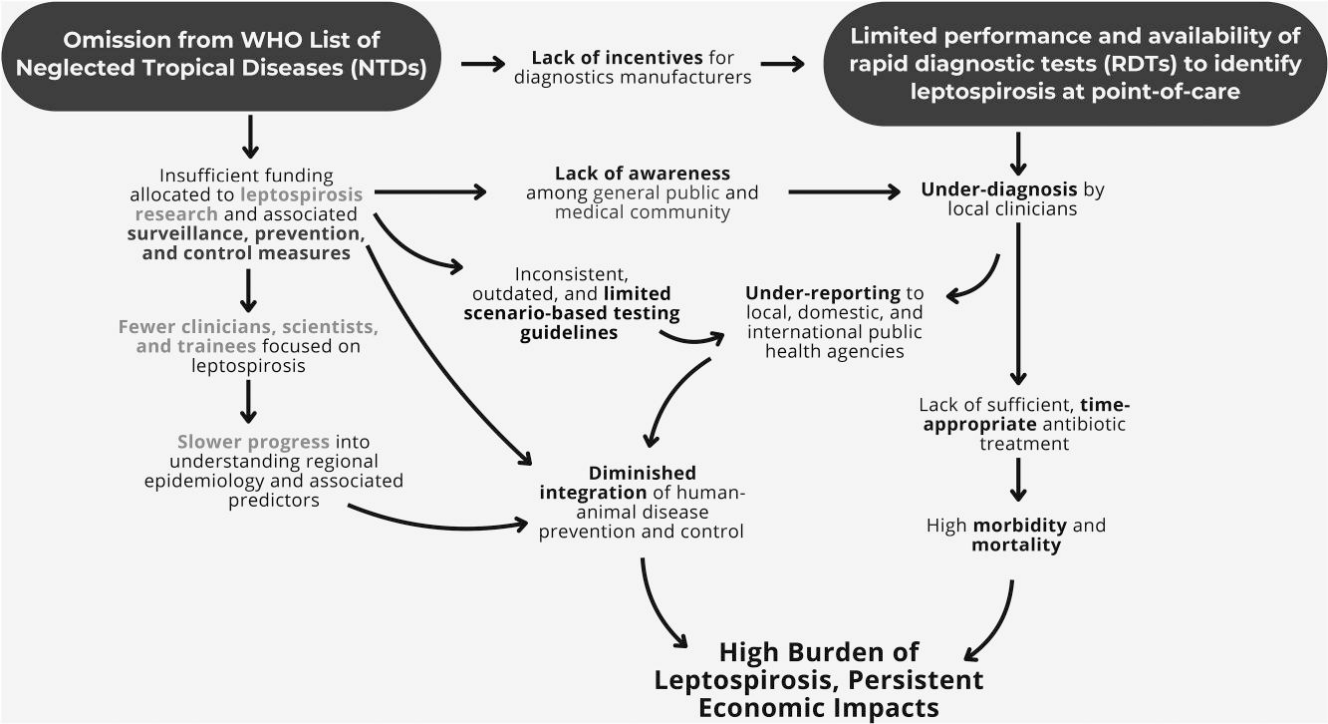
Pathways of neglect and their impacts on leptospirosis. Underrecognition of leptospirosis as a neglected tropical disease results in insufficient funding for research, surveillance, prevention, control, and diagnostics, which reduces awareness, limits access to diagnostics, and results in therapeutic delays that produce a higher burden of infection.

Early leptospirosis, when treatment is most effective, is frequently difficult to distinguish from other acute febrile illnesses such as dengue and malaria, posing a significant barrier to improving healthcare outcomes. Underrecognition and underdiagnosis lead to an increased risk of severe illness and lack of awareness of outbreaks. Leptospirosis exemplifies healthcare inequity, predominantly affecting underserved communities in tropical regions, which often lack essential healthcare resources and sanitation infrastructure [77]. Figure 1 summarizes the pathways of neglect and their impact on the burden of leptospirosis.

## AMENABILITY TO INVESTMENTS IN IMPROVING HEALTHCARE OUTCOMES

Leptospirosis urgently requires investments in prevention, diagnosis, and treatment strategies to break the cycle of underrecognition, underdiagnosis, and underreporting that lead to a high burden of disease. A comprehensive One Health approach that integrates collaborative research, reporting, and guidelines to improve human and veterinary health diagnostics and surveillance systems would significantly advance the body of knowledge about leptospirosis disease, eco-epidemiology, public health preparedness, and interventions where they are most needed [9, 28, 71, 78]​.

More effective surveillance and control strategies could identify early outbreaks, track zoonotic transmission, and pinpoint environmental hotspots where both humans and animals are at risk [79]. In places where surveillance has been strengthened, data collection has led to a better understanding of the disease and the implementation of effective control measures that other countries can replicate [80]. Coordinated surveillance and reporting allow for faster response times, improved detection, and enhanced community and professional awareness, thereby reducing transmission through behavioral changes, particularly in high-risk areas like urban slums and rural agricultural regions [81]. Additionally, it promotes cross-sector collaboration, ensuring that prevention and intervention strategies are more effective and tailored to the dynamic nature of disease transmission in both human and animal populations.

Primary healthcare systems at the village level play a crucial role in disease prevention and health promotion, as healthcare workers in these settings are often the first to encounter individuals with early symptoms of leptospirosis. Raising awareness among healthcare providers can improve early recognition, ensure timely referral to higher level care for confirmatory diagnosis, and facilitate prompt treatment, ultimately reducing the burden of severe disease and organ damage. Primary care physicians must often rely on clinical characteristics, signs, and symptoms to identify patients suspected of leptospirosis. Faine's criteria or the WHO score is a diagnostic tool developed in 1982 to help clinicians make a presumptive diagnosis of leptospirosis [82]. It combines clinical data, epidemiological factors, and laboratory findings to assess the likelihood of leptospirosis. Faine's criteria have undergone modifications to enhance the diagnostic process by refining clinical symptoms and incorporating new, more accessible laboratory techniques [16, 83–86].

For hospitalized patients presenting with severe symptoms, an on-admission diagnostic score was developed using inpatient data from hospitals in northeastern and southern Thailand. This score consists of 7 variables, including clinical indicators and routine laboratory tests available in all hospitals, providing a practical tool for early presumptive diagnosis in severe cases of leptospirosis [17]. It can be applied at the point of care, enabling initial assessments while awaiting confirmatory laboratory results. This scoring system has been integrated into Thailand's leptospirosis clinical guidelines by the Department of Disease Control, Ministry of Public Health in 2021 [87] to improve the early detection and management of severe leptospirosis cases across hospitals and rural health centers. The score also raises awareness of leptospirosis in community hospitals because it is practical and easy to use in general practice while awaiting laboratory results. Although these clinical diagnostic algorithms are promising, validation studies are recommended to determine accuracy and effectiveness to improve patient outcomes in diverse clinical settings. This will help ensure the tool's reliability and clinical utility across different healthcare environments.

Rapid diagnostic tests are urgently needed to deliver accurate results within 1 hour, require minimal training, maintain stability during storage, and cost little to manufacture and deploy. Strategic funding initiatives are needed to overcome these market barriers and improve access to such diagnostic tools. With adequate funding, point-of-care diagnostics could become cost-effective and more widely produced, ultimately making them accessible to patients in high-risk regions. For example, a Thai research group developed a point-of-care polymerase chain reaction-based detection system using RPA-CRISPR/Cas12a on blood or urine samples, with highest sensitivity during first week of infection, making this method an excellent tool for early detection [88]. Loop-mediated isothermal amplification technology is an approach that is compatible with point-of-care testing platforms and has shown good performance for detection of leptospires in various clinical sample types [89, 90]. The BioFire Global Fever Panel uses whole blood to test for malaria, leptospirosis, chikungunya, and dengue, accurately detecting *Leptospira* spp. in 93.8% of independently confirmed samples [91].

Serologic testing (eg, immunoglobulin M [IgM] rapid diagnostic test (RDT), IgM enzyme-linked immunosorbent assay) can be used for diagnosis once IgM antibodies are developed but are often negative in the first week of infection. Development of serologic RDTs detecting IgM or IgM/IgG antibodies have improved considerably, indicating their potential to fill the gap in clinical case detection for global application [79, 92, 93]. A prospective study evaluated the accuracy of 5 commercially available IgM-RDTs and reported marked differences in sensitivities by test and that the highest sensitivities (70%–80% for the best tests) were reached 4–6 days after fever onset, while specificity remained stable [94]. Clinicians are advised to combine RDT results with exposure history, symptom onset, and clinical presentation to interpret patients' posttest probability. This highlights the need for a combined approach using point-of-care diagnostics that detect the pathogen's DNA and IgM detection to maximize sensitivity and reduce the likelihood of misdiagnosis in the early stages of infection [95].

Educating healthcare providers on the importance of initiating antibiotics early, even before confirmatory diagnostic results are available, is key to preventing the progression to severe disease, including organ failure. Leptospirosis can be effectively treated with a range of low-cost antibiotics and there are no reports of antibiotic resistance [96]. Doxycycline is considered first-line treatment for mild-moderate suspected cases, with penicillin (intravenous) recommended for severe cases (ie, organ failure) or in pregnant women [2]. Both antibiotics reduce the severity and duration of the disease, with doxycycline often preferred because of its broad availability and ease of administration. Azithromycin, dosed orally, is also an alternative for patients unable to tolerate doxycycline or penicillin [2]​, with additional medications such as tetracycline, ceftriaxone, and cefotaxime occasionally used in specific clinical scenarios. Each of these treatments are widely available and are included on the WHO essential medicines list [97].

**Figure 2.**
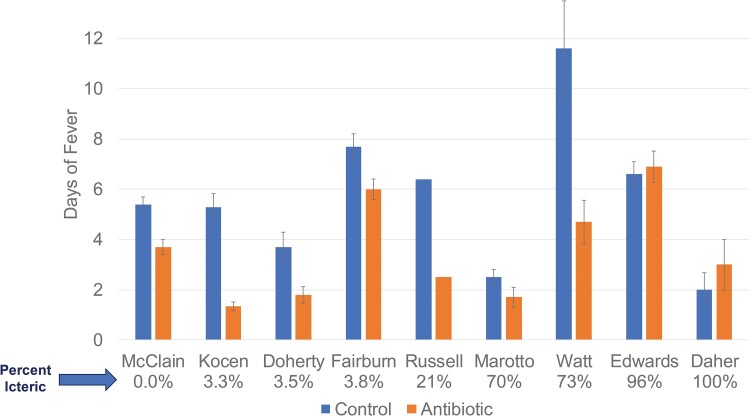
Controlled studies comparing fever duration ± antibiotic therapy for leptospirosis. Controlled studies of antibiotic therapy for leptospirosis arranged by percent of icteric patients at time of enrollment. All studies showed reduced days of fever except those with ≥96% icteric patients. References provided in the text.

The ideal timeframe for treatment is within the first 6 days of symptom onset, with greatest reductions in fever duration and related complications occurring when antibiotic treatment is initiated within 2 days of symptom onset [98–103]. Pioneering research into this involved a randomized, placebo-controlled trial conducted at a US Army jungle training center in Panama [98], where McClain et al. found that doxycycline 100 mg twice a day initiated, on average, 2 days after symptom onset and before the onset of renal failure or jaundice, reduced postantibiotic fever duration from 5.4 days to 3.7 days, and, most critically, prevented the onset of renal failure or jaundice [98]. Five additional randomized controlled trials, involving almost 300 patients with confirmed leptospirosis, demonstrate significantly earlier resolution of febrile symptoms when either penicillin, ampicillin, or oxytetracycline were initiated (average treatment onset 6 days after onset of symptoms) (Figure 2). In contrast, reports that failed to show an impact of antibiotics involved patient populations consisting almost entirely of patients with late leptospirosis and advanced organ dysfunction [104, 105], when response to antibiotics would be highly unlikely. The association of early treatment with improved outcomes in leptospirosis is a fundamental principle of infectious diseases.

Early diagnosis is important to implement therapeutic interventions that are not limited to antibiotics. Supportive care including hydration and correction of electrolyte imbalance during the early, nonoliguric phase of kidney involvement can prevent more severe renal impairment [106, 107]. In cases in which oliguric renal failure occurs, early initiation of peritoneal dialysis or hemodialysis can be lifesaving [108]. In a comparative study of patients with leptospirosis in São Paulo, Brazil, reducing the time from admission to initiation of hemodialysis from 27 to <5 hours was associated with a reduction in mortality from 66.7% to 16.7% [109]. Similarly, in a cohort of intensive care unit patients on the island nation of Réunion, mortality fell from 20% to 6% through the implementation of early and intensive management [110]. Establishing efficient referral systems that ensure patients with severe disease are transferred to tertiary care centers promptly will be essential in reducing mortality associated with leptospirosis.

Early diagnosis and treatment could reduce the number of DALYs lost. A cost-effectiveness analysis of test and treat strategies revealed the benefit of implementing diagnostic testing to improve outcomes while decreasing the overuse of antibiotics [111]. Improved diagnostics and early intervention would be cost-effective, leading to better patient outcomes and reducing healthcare costs associated with hemodialysis, extended intensive care unit stays, and disability-related employment loss.

Vaccination of livestock and pets can reduce shedding and/or infection and limit their role as reservoirs, while improving sanitation and rodent control helps to minimize environmental contamination. Public education on safe animal handling and waste management can further protect human health by reducing exposure to this zoonotic pathogen across interconnected animal populations. Public health infrastructure improvements, including addressing sanitation deficiencies such as open sewers, waste removal, and floodwater management address key risk factors for leptospirosis transmission [73]. Studies have demonstrated the cost-effectiveness of sanitation interventions [112]. Brazil's efforts in improving sanitation infrastructure have directly reduced leptospirosis transmission in vulnerable populations [81].

## CLOSING REMARKS

**Figure 3.**
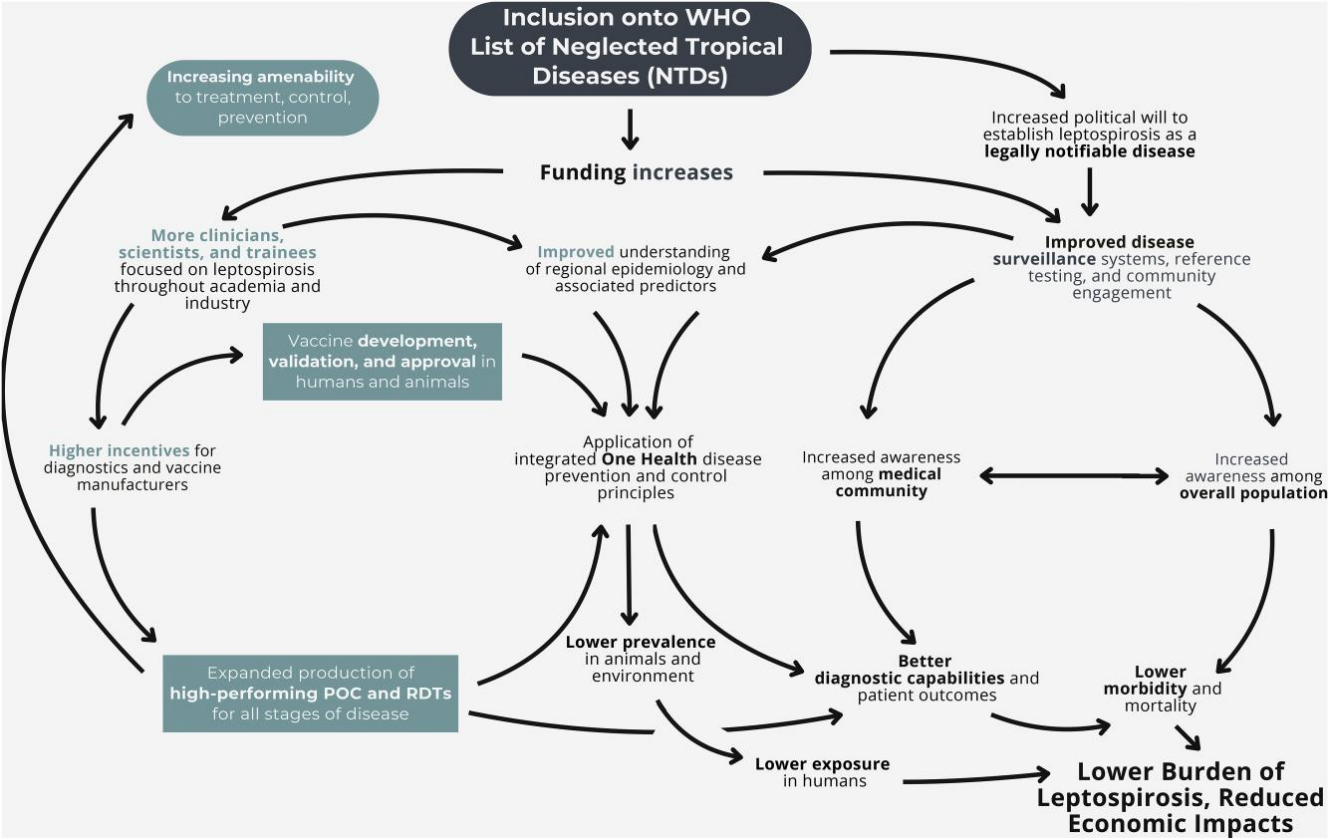
Pathways of investment and their impacts on leptospirosis. Recognition of leptospirosis as a neglected tropical disease results in increased investment in community engagement, physician awareness, improved surveillance, integrated One Health disease prevention and control, access to rapid diagnostics, and early treatment—all of which reduce the disease burden.

As summarized in Figure 3, recognizing leptospirosis as a neglected tropical disease is critical to boosting investment in research, development, and public health initiatives aimed at improving patient outcomes. It is important to advocate for leptospirosis to be included in the WHO's NTD list and those of other public health agencies because many funding organizations use these lists for disease prioritization. Increased funding is essential for research focused on understanding the life cycle of leptospiral spirochetes and other waterborne zoonotic pathogens across diverse ecosystems, as well as for advancing early diagnostic tools and expanding access to these diagnostics in endemic areas. Employing a One Health approach, which considers the interconnections between human, animal, and environmental health, is particularly valuable for an integrated surveillance system and for managing and understanding the transmission dynamics of leptospirosis from animals to humans. Climate change, with its effects on flooding and ecological disruption, combined with socioeconomic factors such as poverty and displacement, is likely to exacerbate the incidence of leptospirosis. Strategic investment in targeted research, public health campaigns, and rapid point-of-care diagnostics can raise awareness, facilitate earlier detection, and improve treatment outcomes, thereby reducing the overall burden of this disease.

## Notes

***Acknowledgments.*** The authors thank Lauren Kerr for improving the manuscript and contributing to Figures 1 and 3.

***Financial support*.** Work on this review was supported by the Swiss National Science Foundation (A.D.), by the Center for Global Health and Social Responsibility, University of Minnesota (C.M.-Z.), Climate Change and Health Research Coordinating Center (C.M.-Z.), National Institutes of Health/National Institute of Allergy and Infectious Diseases (NIH/NIAID) R01 AI183353 (U.L., N.S., and D.A.H.), and NIH/NIAID P01 AI168148 (U.L., N.S., M.P., and D.A.H.) and Institute Pasteur (M.P.).

***Patient consent statement.*** This manuscript is a review that does not include clinical or patient data, thereby negating the need for patient consent.
